# Loneliness and its correlation with self-care and activities of daily living among older adults: a partial least squares model

**DOI:** 10.1186/s12877-024-05215-7

**Published:** 2024-07-20

**Authors:** Nazanin Masoudi, Ehsan Sarbazi, Hassan Soleimanpour, Mehdi Abbasian, Masouma Ghasemi, Zahra Rostami, Hosein Azizi, Maryam Soleimanpour

**Affiliations:** 1 Student Research Committee, Tabriz University of Medical Sciences, Tabriz, Iran; 2 Department of Statistics and Epidemiology, Tabriz University of Medical Sciences, Tabriz, Iran; 3 Road Traffic Injury Research Center, Tabriz University of Medical Sciences, Tabriz, Iran; 4 Medical Philosophy and History Research Center, Tabriz University of Medical Sciences, Tabriz, Iran; 5 Student Research Committee, Zanjan University of Medical Sciences, Zanjan, Iran; 6 Women’s Reproductive Health Research Center, Tabriz University of Medical Sciences, Tabriz, Iran; 7 Clinical Research Development Unit of Tabriz Valiasr Hospital, Tabriz University of Medical Sciences, Tabriz, Iran

**Keywords:** Loneliness, Self-care, Activities of daily living, Older adults, Epidemiology

## Abstract

**Background:**

The growing elderly population worldwide is accompanied by an increased disrupting daily activities and self-care. Neglecting the multifaceted needs of the elderly can lead to detrimental effects such as loneliness or social isolation, threatening healthy aging. Self-care is a key strategy to enhance daily functioning and mitigate feelings of loneliness among the elderly. This study was conducted with the aim of investigating the feelings of loneliness and its relationship with self-care and Activities of Daily Living (ADL) among the older adults of Tabriz city.

**Methods:**

In this observational cross-sectional study, we engaged 315 older adults using a simple random sampling. Participants were selected randomly from Iran’s Integrated Health System (IIHS) framework. Three questionnaires including de Jong Gierveld Loneliness Scale, Persian version of self-care scale, and ADL-Katz were used for data collection. The Partial Least Squares and Spearman’s correlation were used to investigate the relationships between demographic characteristics, loneliness, self-care, and ADL.

**Results:**

The sample comprised 315 respondents 51.1% were female, 49.5% had a middle school literacy and 86% were married. A significant negative relationship was observed between loneliness and self-care (*P* < 0.001 and *r* =-0.311). Demographic characteristics, including age and marital status, were found to negatively moderate the relationship between self-care (path coefficient − 0.07, *P* = 0.044) and positively moderate the relationship with loneliness (path coefficient 0.29, *p* < 0.001). ADL was positively associated with self-care (path coefficient 0.41, *p* = 0.046) and also a direct and significant relationship was observed between ADL and daily self-care (*P* < 0.001 and *r* = 0.335).

**Conclusion:**

This study underscores the complex interplay between loneliness, self-care, and ADL. It highlights the need for interventions that address emotional health and daily living skills as part of comprehensive self-care strategies. Further research is needed to explore these relationships in more detail and to develop targeted interventions for different demographic groups.

## Introduction

Aging is defined as the progressive deterioration of the structure and function of body organs over time and is associated with biological and physiological changes [1, 2]. Nowadays progression and developments in education, economic welfare, nutrient improvement, general health condition, and medical care have made people live longer than in the past [3–6]. Due to increased life expectancy and reduced mortality, the world’s population is growing old and the number of older adults is increasing [7]. The world’s older adults population (over 60 years old) is anticipated to reach more than 2 billion by 2050 [8].

According to WHO reports, through 2030, one in six people in the world may well be aged sixty years or over [9] and, by 2050, the global population of aged people is going to be 2.1 billion [9]. In Iran, the population over the age of sixty-five is anticipated to grow to 11% and 17% of the overall population in 2036 and 2051, respectively [10–12]. Reductions in fertility and mortality are the main causes of the aging of Iran’s population. The rapid and sharp decline in fertility rates over the past three decades, along with a significant increase in life expectancy, will contribute to Iran’s quick growth of older people size [13, 14].

With the increasing age of people, and as their ability to perform daily tasks decreases, they require special attention from health systems, with a special focus on self-care [15]. Self-care is a critical principle in old age and features favorable consequences in providing, maintaining, and promoting the health of the older adults [16, 17]. In addition, self-care is one of the effective mental interventions and the implementation of the its program had a tremendous impact on improving the ability of the aged and improving health behaviors [18, 19].

Although feelings of loneliness are found in all age groups, it’s more common in old age [20]. In addition, studies on the older adults have shown that most of them feel lonely and find it a painful and excruciating feeling [21, 22]. Recent studies in the United States (US) indicate that between 17% and 57% of people who experience loneliness, mainly suffer from anxiety, depression, and dementia [23], and additionally there is a significant correlation between loneliness and depression in the older adults [24].

loneliness is associated with cognitive decline, social isolation, despair, and inability to perform basic daily activities that can potentially compromise their health [21]. Loneliness in older adult’s depends on many factors and it can play an etiological role in causing physical and health problems among them and not only threatens a person’s sense of mental well-being but may also cause self-destruction. The studies conducted in relation to the feeling of loneliness in the older adults and its effect on self-care show that loneliness is significantly associated with subjective well-being and self-care [25, 26]. Loneliness has a negative effect on the quality of life in old age and can reduce the level of self-care by decreasing the quality of life [21, 27]. Therefore, diagnosis of loneliness can be effective in maintaining the health of the older adults, it is necessary to be aware of the consequences of loneliness and its effective effects on the health of aged people.

The capability of performing daily life activities reflects a critical element of functional independence in the older adults and with age it becomes tough to carry out impartial activities of life in old age [28]. Activities of Daily Living (ADL) are basic activities that involve taking care of yourself and your body, including personal care, mobility, and eating. It needs to be stated that impaired self-care ability ends in the lack of ability to carry out ADL, and due to the fact most older adults suffer from chronic diseases, the more their self-care ability is increased, the better they can cope with daily activities [29, 30].

However, far too little attention has been paid to importance of self-care, ADL and feelings of loneliness among older adult’s people as a vulnerable group in Iran. The aim of this study was to investigate the status of the self-care and their relation to ADL and loneliness among the older adults in Tabriz, Iran.

## Methods

### Study population, study design and setting

The current study is an observational cross-sectional analysis of 315 older adult residents of the Tabriz city. Tabriz is the capital of the East Azerbaijan province and Iran’s fourth-largest city overall, Tabriz is located in northwest Iran and has a population that is older than 60 years old in excess of 10% of the total. The largest urban area and economic center in Iran’s northwest is Tabriz. At the beginning of 2021, Tabriz had a total population of 1,603,799, including 170,135 people over the age of 60, 85,387 of whom were males.

### Sampling methods

The sampling method was Simple Random Sampling Using sample size calculation in medical studies formula $\:\:n=\frac{{Z}^{2}P\left(1-P\right)}{{d}^{2}}$[31] and based on the study of Novrouzi et al. [32]. , *p* = 0.65, margin of error = 5.27%, and z = 1.96 the sample size was nearly 315 people. Twenty samples were selected from each of the 16 health centers in Tabriz, resulting in a total of 320 older adults. This exceeds the minimum sample size requirement of 315. The study subjects were then randomly selected from this pool of older adults by Iran’s Hospital Information System (HIS) randomly [33, 34]. The samples were given a written consent form to complete at the end of interviews.

### Inclusion and exclusion criteria

All of the older adult participants in the study were who were over 60 and willing to take part. Exclusion criteria were hearing or mental problems.

### Self-care

Self-Care tool’s items were 40, the questionnaire comprised 40 items. Each item was evaluated using a four-point Likert scale. The response options for this scale included ‘often’, ‘sometimes’, ‘rarely’, and ‘never’. Furthermore, it representing the five main factors with acceptable validity of the contents. Together, these five factors explained 79.93% of the variance. The first factor, physical self-care, had Cronbach’s α of 0.746. Daily self-care was the second factor, with a factor of 0.746, psychological self-care was the third, with a α of 0.845, social self-care was the fourth, with a factor of 0.831, and the dimension of self-care during illness was the fifth, with a factor of 0.905. The questionnaire’s internal consistency was 0.864 [35].

### Activities of daily living

We used the Persian version of ADL-Katz which was validated for Iranian older adult people [36]. The total ADL-Katz score lies on an ordinal scale ascending from 0 to 6, where 6 points is considered as independency and 0 points are measured as a full dependency. Katz’s index of ADL summarizes six functions that are necessary for self-care composed of decreasing ability or difficulty in bathing ability, dressing, going to the toilet, movement, continence, and feeding.

### Loneliness scale

The Persian version of the 6-item de Jong Gierveld loneliness scale had an acceptable content validity (CVI = 0.874). It includes two factors: emotional loneliness and social loneliness. The reliability of the scale was accepted by the intra-class correlation coefficient and the α coefficient [37]. The internal consistency of the three scales was assessed using α which ranged 0.90–0.95.

### Statistical analysis

Descriptive statistical methods, mean, Standard Deviation (SD), frequency, and percentage were used to analyze the data. For screening the differences in basic characteristics between the two groups, the t-test was used for continuous variables and the chi-square test for stratified variables.

### Evaluation of Structural Model

In this study, the main effect and the moderating effect were each analyzed when the structural model was tested. First, the main effects were tested to analyze the effects of each latent variable. To clarify the path of demographic, loneliness, and ADL on self-care, PLS Bootstrap method with 5000 resampling to obtain inference statistics. Inorder to evaluation of validity of the structural model the coefficient of determination (R2 ) goodness of fit (GoF) index, the standardized root means square residuals (SRMR), and the normed fit index (NFI) for assessing the validity of the structural mode were applied [38]. Statistical analyzes were performed by SPSS software version 20, and the significance level was considered 0.05. The Partial Least Squares (PLS) was used to investigate the relationships between demographics, ADL, and loneliness with self-care. SmartPLS v.3.3.6 was used for data analysis.

### Ethics

The study was approved by the Ethical Committee of the Tabriz University of Medical Science (https://ethics.research.ac.ir/IR.TBZMED.REC.1400.1225).

## Results

### Study participant characteristics

Characteristics of the sample are presented in Table 1. Among participants 47.9% were male and 51.1% were female. 66% of respondents were in 60–69 years old. 86% of participants had a married status and 49.5% had a middle school education level. Mean, and standard deviation of loneliness, self-care, and ADL among the study participants were presented in Table 2.

**Table 1:** Study population characteristics (*N* = 315)

Variable	Categories	Number	percent
Age(years)	60–69	209	66.3%
	70–79	80	25.4%
	80+	26	8.3%
Gender	Male	151	47.9%
	Female	164	52.1%
Education	Elementary	105	33.3%
	Middle	156	49.5%
	High	42	13.3%
	College or higher	12	3.8%
Marital status	Married	271	86%
	Widow/widower	44	14%

**Table 2:** Cronbach’s alpha, mean, and standard deviation of loneliness, activities of daily living, and self-care among the study participants

Constructs	Items	Mean	SD	Cronbach’s α
Loneliness	social loneliness	0.38	0.79	0.93
	emotional loneliness	0.37	0.71	0.90
Self-care	Emotional	23.56	1.83	0.90
	disease	39.44	3.09	
	social	33.48	2.74	
	physical	27.64	1.90	
	daily	19.66	1.63	
ADL		5.44	1.48	0.95

SD: Standard Deviation, ADL: Activities of Daily Living

### Relationships between loneliness, self-care, and ADL

**Fig. 1:**
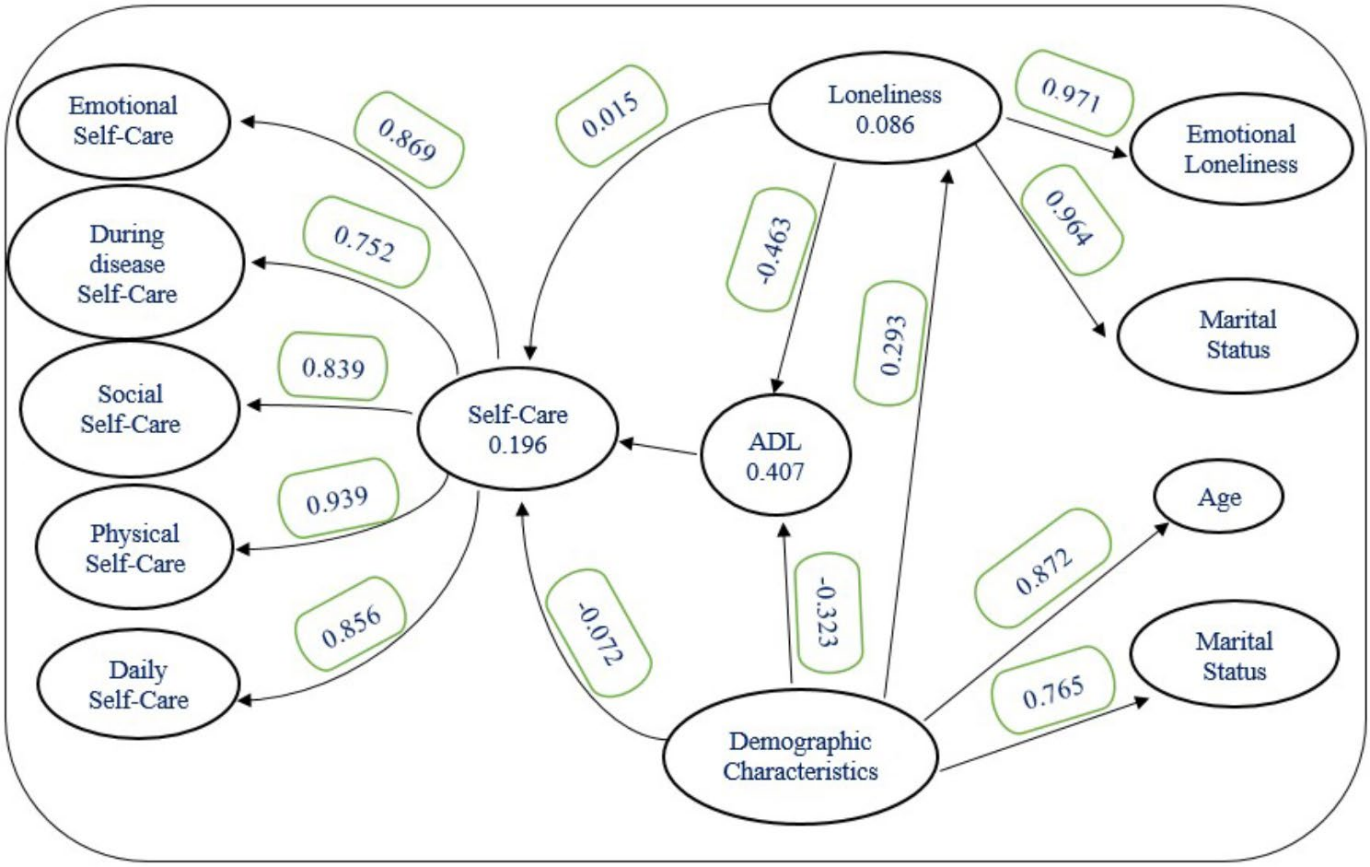
Path model of demographic status, loneliness, and Activities of Daily Living (ADL) on self-care among elderly people

The direct and indirect effects can be seen in Table 3. Loneliness (path coefficient 0.01, *p* = 0.880) did not show a significant relationship with self-care directly. Loneliness (path coefficient − 0.19, *p* = 0.047) demonstrated an indirect significant relationship with self-care directly. Loneliness (path coefficient − 0.46, p = < 0.001) shows a significant direct relationship with ADL. Demographic (path coefficient 0.29, p = < 0.001) show a significant direct relationship with loneliness. The demographics had a negatively associated with ADL (path coefficient − 0.32, *p* < 0.001), ADL was positively associated with self-care (path coefficient 0.41, *p* = 0.046) and negatively moderated the relationships between loneliness and self-care (path coefficient − 0.19, *P* = 0.047) and loneliness (path coefficient − 0.14, *p* = 0.001) negatively moderated the relationships between demographic and ADL. Model fitting parameters were SRMR = 0.07 and NFI = 0.80 and R2 = 0.41. The Path model of demographic status, loneliness, and ADL on self-care among older adult people relationships are shown in Fig. 1.

**Table 3:** Direct and indirect effects of association among loneliness, demographic variables, self-care, and activities of daily living

	Path way	Path coefficient	*P* value
Direct effect*	Demographic → loneliness	0.29	< 0.001
	loneliness → self-care	0.01	0.880
	loneliness → ADL	-0.46	< 0.001
	Demographic → self-care	-0.07	0.441
	Demographic^*^ → ADL^*^	-0.32	< 0.001
	ADL → self-care	0.41	0.046
Iindirect effect^**^	lonekines → ADL → self-care	-0.19	0.047
	Demographic → loneliness → self-care	0.00	0.886
	Demographic → loneliness → ADL	-0.14	0.001
	Demographic → lonelines → ADL → self-care	-0.06	0.084
	Demographic → ADL → self-care	-0.13	0.073

*Demographic variables such as age and marital status, ADL: activity of daily living, *The effect of demographic variables, ADL, and feelings of loneliness in a direct way, ** Hierarchical and indirect-way effects on ADL and self-care

The relationship between self-care, daily activities, and feelings of loneliness in elderly individuals, as indicated by the Spearman’s correlation coefficient, is presented in Table 4.

**Table 4:** Spearman’s correlation coefficient between self-care, activities of daily living and loneliness in the older adults

	physical SC	daily SC	Emotional SC	social SC	during disease SC	self-care
ADL	0.251^*^	0.335^*^	0.183^*^	0.200^*^	0.265^*^	0.255^*^
emotional loneliness	− 0.290^*^	− 0.205^*^	− 0.208^*^	− 0.112	− 0.105	− 0.311^*^
social loneliness	− 0.276^*^	− 0.205^*^	− 0.175^*^	− 0.079	− 0.102	− 0.297^*^

SC: Self-Care, ADL: Activities of Daily Living, *P < 0.001

## Discussion

The aim of this study was to investigate the feelings of loneliness and its relationship with self-care and ADL among a sample of 315 older adults people living in Tabriz, Iran. Loneliness, often experienced by older adults, is a sense of isolation and constant absence of others, exacerbated by the stress of losing spouses and loved ones.

In our study, the feeling of loneliness in the older adults had an inverse and indirect relationship with self-care. In 2021, Shamlou et al. conducted research aimed at investigating the relationship between the loneliness of the older adults and self-care ability, and the results indicated that more than 70% of the older adults experienced a feeling of loneliness and reported a direct relationship between loneliness and the self-care ability of the older adults [25]. The discrepancy in the findings of this study could be attributed to the variation in the questionnaires used in these two investigations. Furthermore, the advent of the coronavirus pandemic [39] has altered perceptions of self-care, particularly among the older adults. This shift could explain why loneliness does not directly influence self-care. Moreover, conducting more in-depth research could provide a more nuanced understanding of the relationship between these two factors.

In a study conducted by Lee et al. in 2020, which investigated the demographic, health, and psychosocial correlations of loneliness in older adults, the results indicated that marital status was significantly linked to higher loneliness. Similarly, our study observed a direct and significant relationship between loneliness in older adults and demographic information (such as age and marital status). The results of these two studies were consistent [40].

Like the results of our study, in the study of Tomioka et al. in 2016, the results showed that the lonely older adults are more likely to experience a decrease in ADL [41]. It’s important to recognize that feelings of loneliness can persist in individuals despite the presence of family and friends. The unique nature of each individual’s experience with loneliness can pose challenges in implementing standardized interventions. Consequently, it is imperative to adevise targeted strategies to address these issues, with the aim of enhancing the daily functionality of older adults.

In this regard, in 2018, Jing et al. conducted interventions, such as cognitive therapy, to alleviate feelings of loneliness in older adults. Their results indicated that these interventions not only reduced feelings of loneliness but also improved the level of Activities of Daily Living (ADL) [42]. It’s important to note that the scoring system used in their study is different from ours. In their study, a higher score indicated a greater degree of dysfunction, whereas in our study, a higher score indicates a greater ability to perform ADL. We used the Persian version of the ADL-Katz scale, which was validated for Iranian older adults [36].The total ADL-Katz score lies on an ordinal scale ascending from 0 to 6, where 6 points indicate independence and 0 points indicate full dependency.

A study conducted in Turkey to investigate the feeling of loneliness and its dependence on ADL showed that with the increase in the factors affecting the loneliness of the older adults, performing ADL will be accompanied by many difficulties and challenges [26], which confirms the results of our study.

A study conducted in 2022 by Xiao et al. in China aimed to explore the intermediary roles of loneliness and sleep quality among the elderly Chinese population. The findings of this study revealed a significant relationship between ADL and loneliness. In fact, nearly 24% of the total impact on the psychological distress experienced by the elderly was attributed to this relationship. Our study similarly found a negative correlation between ADL and loneliness. Xiao et al. gathered their data through structured questionnaires and interviews with the elderly, akin to our methodology. However, they employed a multi-stage stratified cluster sampling, which resulted in a larger sample size of approximately 3,300 individuals [43].

To investigate the relationship between ADLs, demographic characteristics and loneliness on self-care, PLS analysis was used and the results show that demographic information (age and marital status) has an inverse relationship with daily life activities. In this regard; in a study in China investigated the impact of changes in ADL by demographic information (marital status and health care) of the older adults, and the results of this study showed that the older adults who were married were less likely to decrease ADL compared to their counterparts [44].

Ultimately, a study among the Brazilian older adults, determined gender differences in the incidence of disability in ADL and the results of this study after adjustment of socio-economic indicators show that older adults women are more disabled and vulnerable than men [45]. Therefore, according to the results of the studies and the differences between the two sexes and the impact of demographic factors on ADL; the results of the present study, which showed an inverse relationship between the indicators of age, gender and marital status of the older adults with daily life activities; Is consistent.

### Limitations

The limitations of this research are that the information’s were completed only by older people who were literate and had the ability to complete the questionnaire, which can cause potential selection bias. In addition, like many cross-sectional studies; It is better to investigate the results with the help of longitudinal studies in the future.

## Conclusion

Given the profound impact of loneliness on the elderly population and the demonstrated effectiveness of interventions in this area, we recommend that healthcare systems and policymakers prioritize the planning, design, and promotion of interventions. These could include increasing social events for the elderly, implementing group therapy, and forming sports groups, all of which could contribute to reducing feelings of loneliness among the elderly. Our study’s findings highlight a positive correlation between ADL and self-care in the elderly, emphasizing the importance of enhancing ADL performance due to its significant influence on crucial health aspects such as self-care. However, future research is needed to further explore self-care among the elderly in various societies, its impact on health, loneliness, and the effectiveness of interventions aimed at reducing it. It’s important to note that our study was conducted exclusively among literate elderly individuals.

## Data Availability

The datasets used and/or analyzed during the present study are available from the corresponding author upon request.
